# Acute Renal Failure due to Organophosphate Poisoning: A Case Report

**DOI:** 10.7759/cureus.1523

**Published:** 2017-07-27

**Authors:** Rizwan Zafar, Kamran Munawar, Adeel Nasrullah, Shujaul Haq, Haider Ghazanfar, Abu Baker Sheikh, Ali Y Khan

**Affiliations:** 1 Department of Internal Medicine, Shifa International Hospital; 2 Internal Medicine, Shifa College Of Medicine; 3 Internal Medicine, Newark Beth Israel Medical Center; 4 Department of Internal Medicine & Infectious Diseases, Shifa International Hospital

**Keywords:** organophosphate, poisoning, acute renal failure, cholinergic crisis, respiratory distress

## Abstract

Organophosphate (OP) poisoning is a commonly seen condition in many countries. OP poisoning classically presents with symptoms of cholinergic excess. It can rarely affect other organ systems but when it does, it can worsen a patient's overall prognosis. We present a case of a 23-year-old man with an extremely rare case of acute kidney injury due to OP, who was successfully treated with a combination of hemodialysis, atropine and pralidoxime days after OP poisoning with reservations on the aging process.

## Introduction

Throughout the world, an estimated 3 million people are exposed to organophosphates (OP) or carbamates yearly, with up to 10% (300,000) fatalities [1]. OP poisoning is common in developing countries like Pakistan and has been used for intentional self-poisoning. In Pakistan, the exact numbers of cases or fatalities are difficult to calculate due to an unavailability of databases or a poison surveillance system [2]. Poisoning occurs mostly by voluntary ingestion, inhalation or by absorption through the skin. OP poisoning may manifest acutely with the cholinergic crisis, respiratory distress, and intermediate syndrome or with delayed toxicity.

OP acts by blocking the activity of acetylcholinesterase thus stimulating cholinergic as well as nicotinic receptors. Cholinergic effects can be reversed by atropine but for most neurological manifestations, which are mediated through nicotinergic effects, oximes are needed. Recommendations are to administer these oximes as soon as possible with no benefit after 13 hours [3].

OP can have an effect on other organ systems which although rare, can worsen the presentation and prognosis of the patient. One of the organs affected is the kidney. Although the exact mechanism of acute kidney injury (AKI) is unclear, numerous hypotheses have been proposed. Death can occur as a result of multiple organ distress syndrome (MODS) or renal failure itself [4].

We describe a case of OP ingestion, presenting with low Glasgow Coma Scale (GCS) and AKI, an extremely rare presentation of OP poisoning which was successfully treated by hemodialysis, atropine, and oximes based on clinical judgment.

## Case presentation

A 23-year-old man with no known co-morbid, presented to the emergency department in an intubated state after being referred from another hospital. Four days ago the patient presented to that hospital in the unconscious state with a history of rigors, chills, and 1-2 episodes of diarrhea the previous day. There was no history of documented fever, headache, nausea, vomiting, frothing from the mouth, up rolling of eyes, urinary or bowel incontinence. No history of a drug overdose or ingestion of any suspicious compound was reported by attendants.

Intubation was done at the presentation there due to low GCS. Urine drug screen was negative but it didn’t include OP. He was found to have AKI for which the patient underwent one session of hemodialysis there.

In our emergency room, vital signs revealed blood pressure (BP) of 110/70 mmHg, pulse of 63 beats per minute, and the patient was afebrile. Neurological examination revealed GCS 3/15, pinpoint pupils, with intact corneal reflexes, doll’s eye response and gag reflex. His plantar reflexes were down going on both sides. On chest examination, there was decreased air entry on right side. Laboratory investigations done on admission are shown in Table 1.

**Table 1:** Laboratory investigations at admission. ALT: Alanine aminotransferase; AST: Aspartate aminotransferase; CPK: Creatinine phosphokinase.

Investigation	Value
Haemoglobin	14.20 g/dl
Hematocrit	40.3%
White blood cells	7300/ul
Platelets	249,000/ul
AST	98 units/L
ALT	83 units/L
C-reactive protein	99 mg/l
Serum sodium	151 mEq/L
Serum potassium	3.71 mEq/L
Serum chloride	112 mEq/L
Serum bicarbonate	14.3 mEq/L
Serum creatinine	7.57 mg/dl
Serum blood urea nitrogen	94 mg/dl
Serum CPK	4266 U/L
Serum calcium	7.59 mg/dl

**Figure 1:**
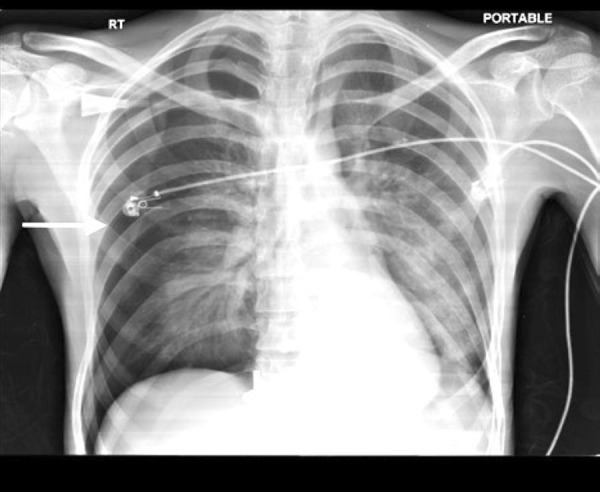
Chest x-ray showing right-sided pneumothorax.

Arterial blood gases (ABG) showed metabolic acidosis. Chest x-ray (CXR) showed iatrogenic right sided pneumothorax (secondary to right subclavian catheter placement at the previous hospital) as seen in Figure 1.

**Figure 2:**
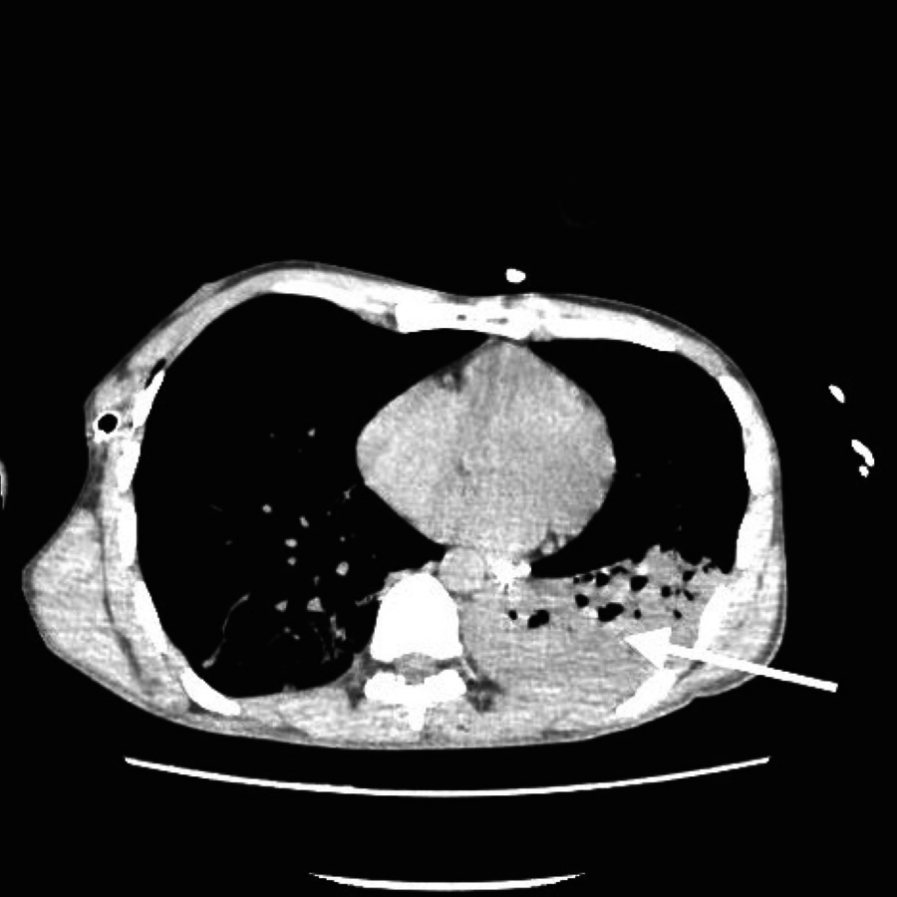
Computed tomography showing large left lower lobe consolidation.

Computed tomography (CT) of chest showed a large consolidation in the left lower lobe of the lung due to aspiration as seen in Figure 2.

Based on history, clinical presentation and laboratory investigations, a differential diagnosis of metabolic encephalopathy, and toxic encephalopathy due to sepsis, possible brain stem diseases and drug overdose were considered. CT of the brain was normal. Treatment was initiated with right chest tube placement for pneumothorax and the patient was given continued ventilator support. Empiric treatment for meningitis, seizure prophylaxis, and 1/2 normal saline was administered for hypernatremia. Lumbar puncture was done and cerebrospinal fluid (CSF) analysis came out negative for meningoencephalitis.

**Figure 3:**
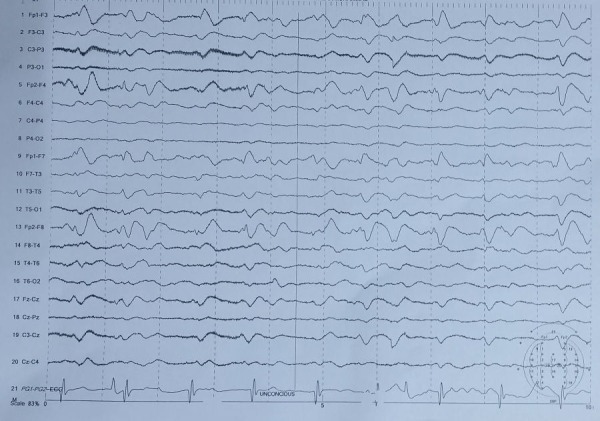
EEG showing severe diffuse encephalopathy of non-specific nature. EEG: Electroencephalogram

Electroencephalogram (EEG) showed severe diffuse encephalopathy as seen in Figure 3.

There was no evidence of dural sinus thrombosis on CT of head with contrast. He was found to have excessive tracheal secretions which were suctioned out. Tracheal culture and sensitivity grew Klebsiella pneumonia and Pseudomonas aeruginosa. Both of them were found to be sensitive to meropenem. A trial of Naloxone was also given but no improvement was seen.

On day 3 of admission, the patient developed bradycardia for which atropine was given multiple times. Atropine improved his heart rate and his pupillary constriction but had no impact on GCS. In addition, he develops hyperreflexia with bilateral well-sustained clonus. He was then administered a bolus of pralidoxime 30 mg/kg followed by an infusion at 8 mg/kg/hr with the strong reservation that “aging” factor might hinder the management with oximes. Injection Atropine 1 mg per hour was also continued.

Significant improvement was seen after six hours of Oximes administration. His GCS improved from no responsiveness to the localization of pain. Tracheostomy was done and after 72 hours of continued treatment, GCS improved to 12/15. Pralidoxime and atropine were continued. Multiple sessions of hemodialysis were performed. He was also given intravenous antibiotics for nosocomial infections. His overall condition improved and he was discharged home in a stable condition. The patient later admitted to ingestion of insecticides over some family dispute. Psychiatry consult was ordered and follow-up was planned.

## Discussion

OP compounds are widely used as insecticides in our agricultural setting. They are easily available and no real care is taken while handling these compounds which have also been used for the purposes of suicides in developing countries [5]. OP poisoning is a common phenomenon which presents classically with signs of cholinergic excess.

OP compounds inhibit acetylcholinesterase by phosphorylating it. Some of the phosphorylated cholinesterases can dealkylate leading to "aged" enzyme which is a non-reversible state [6]. In doing so there is an uncontrolled stimulation of nicotinic and muscarinic receptors by acetylcholine (Ach). Signs of OP poisoning can include diarrhea, excessive salivation, lacrimation, urination, bronchorrhea, bronchospasm, bradycardia, miosis and muscle paralysis. The central nervous system (CNS) does contain nicotinic and muscarinic receptors, and the toxic effects on the CNS include central respiratory depression, agitation, seizures, and coma.

AKI has been seen with OP poisoning. It is seen very rarely and only a few cases have been reported in medical literature. Furthermore, the main mechanism by which OPs can cause AKI has been debated. Different mechanisms have been suggested that can cause AKI. OPs might also cause oxidative stress, direct damage to the renal tubules, rhabdomyolysis, and hypovolemia due to dehydration [4]. One cohort study found that patients with OP poisoning had a 6.17-fold higher risk of AKI compared with the comparison cohort [7].

Different treatment modalities have been tried with OP poisoning ranging from invasive to non-invasive measures. Usually, we start with the removal of the patient from the source, removing contaminated clothing or other items. For severe respiratory depression, ventilatory support is provided [8].

Specific antidotes include atropine and pralidoxime. Atropine inhibits muscarinic receptors and causes a decrease in acetylcholine-induced cholinergic effects. Pralidoxime in contrast to atropine does not affect any specific receptors; rather it acts to regenerate acetylcholinesterase (AchE), which has been rendered non-functional by the OPs. Due to anti-inflammatory effects of IL-10, it has been used in the management of OP poisoning involving organs such as the kidney, liver, and lungs [9].

Our patient was admitted in a comatose state, already intubated and had an oliguric acute renal failure. The cause of his presentation was undetermined but based on clinical judgment, atropine and pralidoxime along with hemodialysis were initiated. After the administration, there was a marked clinical improvement further supporting our suspicion of OP poisoning.

Studies on pralidoxime for the treatment OP have not demonstrated a clear answer whether it is beneficial or not due to multiple factors. Firstly, the type of OP determines how fast the aging process takes place due to different aging half-lives. Furthermore, the dose of OP that the patient has been exposed to also determines response to pralidoxime [10]. We administered pralidoxime as per the WHO guidelines (30 mg/kg bolus, followed by 8 mg/kg/hr infusion) which showed a marked response in our patient.

Further studies are required to determine clear guidelines for the use of pralidoxime and its effectiveness as an antidote for OP poisoning.

## Conclusions

OP poisoning usually presents with typical signs and symptoms of cholinergic excess and rarely can cause acute renal failure. Timely diagnosis and management of this complication confer a favorable prognosis to the patient but if unattended, can lead to a progressively grave clinical course. Additionally, we propose that a trial of oximes be given irrespective of the aging process.
